# Green starch nanoparticles production in situ using α-amylase from a newly isolated *Bacillus subtilis* strain-MA6: statistical designs and characterizations

**DOI:** 10.1186/s12934-025-02812-y

**Published:** 2025-08-18

**Authors:** Mohamed S. Hasanin, Mohamed A. A. Abdella

**Affiliations:** 1 Cellulose and Paper Department, National Research Centre, Dokki, Giza, 12622 Egypt; 2 Chemistry of Natural and Microbial Products Department, Pharmaceutical and Drug Industries Research Institute, National Research Centre, Dokki, Giza, 12622 Egypt

**Keywords:** α-Amylase, Statistical design, Starch nanoparticles, Characterizations

## Abstract

**Background:**

Starch is a carbohydrate polymer, made up of multiple glucose units, connected through glycosidic bonds. Starch nanoparticles (StNPs) are characterized as particles that possess at least one dimension measuring less than 1000 nm, while still being larger than a single molecule, and they have several uses in diverse technological fields. Various studies indicate that synthesizing StNPs through physical and chemical techniques is expensive, requires a lot of energy, and may harm human health and the environment. In contrast, the enzymatic synthesis of StNPs exerts milder impacts on the final products, rendering them more eco-friendly, safe, and healthier. So, amylases can produce StNPs with enhanced solubility, gelation, and viscosity characteristics by hydrolyzing soluble starches.

**Results:**

This study explores the production of starch nanoparticles (StNPs) by α-amylase enzyme in situ from a newly isolated bacterial strain, which was biochemically described, genetically identified, and deposited into the database of GenBank under the designation *Bacillus subtilis* strain-MA6 (accession number: ON840082). The production medium was adjusted by employing statistical optimization of several parameters using the Plackett-Burman design (P-BD) and Box-Behnken design (B-BD) of the response surface methodology (RSM). Optimization of medium parameters using P-BD and B-BD models caused a 14.5-fold increase in α-amylase production. The StNPs were synthesized from bulk starch using three different α-amylase activities. Based on the B-BD results, trial 5 (B-BD/T_5_), trial 7 (B-BD/T_7_), and trial 13 (B-BD/T_13_) were selected for the StNPs characterization using Fourier-transform infrared spectroscopy (FTIR), Dynamic light scattering (DLS), and high-resolution transmission electron microscopy (HR-TEM) analysis. Trial 13 represented the highest α-amylase activity and observed high stability with an average zeta potential of about − 15.1 ± 3.2 mV. Moreover, HR-TEM showed the StNPs as spheres with an average size of about 43 nm.

**Conclusion:**

StNPs were synthesized from bulk starch using the *B. subtilis* strain-MA6 α-amylase enzyme. The concentration of α-amylase plays a role in converting bulk starch to nanosized particles, which affects the stability of the produced nanoparticles and their size. This observation offered an optimistic technique to produce StNPs via a green and eco-friendly process.

**Graphical abstract:**

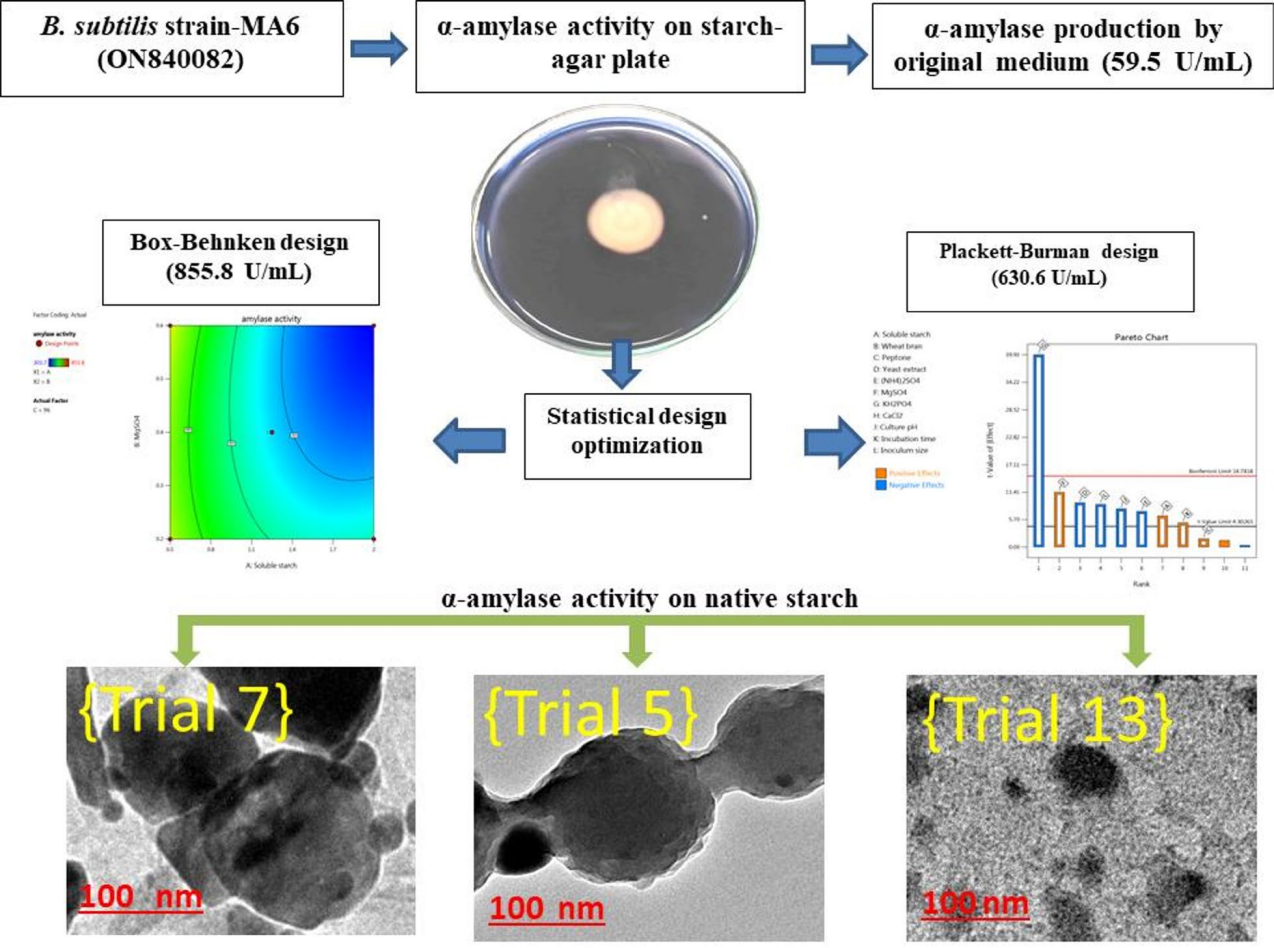

## Introduction

Enzymes are widely recognized as highly efficient biological catalysts that facilitate numerous processes. Furthermore, their use can simplify reactions by minimizing the number of steps and reducing the need for harmful solvents, ultimately lowering the overall cost of the process and making it more ecofriendly [1, 2].

α-Amylase (EC 3.2.1.1) randomly stimulates the hydrolysis of α-(1, 4)-glycosidic linkages within starch molecules to produce distinct oligosaccharides (dextrin, maltotriose, maltose, glucose) of branched and linear chains [3]. Amylases can be obtained from various biological origins, ranging from microbes (fungi, yeasts, and bacteria) to animals and plants [4, 5]. However, microbial α-amylases (particularly from bacterial sources) are superior to other counterparts because they are stable with genetic diversity, high activity, and great tolerance against harsh conditions, simple production, and acceptable costs [5–7]. Various species of the genus *Bacillus* (*B. amyloliquefaciens*,* B. subtilis*, *B. megaterium*,* B. licheniformis*, *B. stearothermophilus*, etc.), which can excrete a large number of extracellular enzymes, are widely known as α-amylase-producing organisms [6, 8]. Further, *Bacillus* species have been exploited for prominent commercial purposes due to a short fermentation period, uniformity, ease of manipulation, safety of processing, and eco-friendly features [6, 7].

α-Amylase has a broad spectrum of industrial applications, including food, textile, animal feed, paper, detergent, pharmaceutical, brewing, baking, and sugar industries [9, 10]. Due to the prominence of α-amylases, obtaining newly isolated bacterial isolates with great potential to produce these enzymes has become an interesting approach for many researchers [9]. Persistent studies are concentrated on developing efficient strategies to optimize medium parameters needed for microbial outgrowth and enzymatic production under submerged fermentation (SmF) [9]. The one-factor-at-a-time (OFAT) optimization process exhausts time and effort. It cannot investigate the combinations between several variables, causing misconstruction of the experiments [9, 11]. The deficiency of the OFAT method can be addressed through the procedure of developed techniques, depending on multiple-factorial design models involving the Plackett-Burman design (P-BD) and Box-Behnken design (B-BD) of the response surface methodology (RSM) [8]. These statistical (mathematical) models lower the number of trials, reduce the cost of bioprocess, simplify the investigation of various variables, illustrate the interactive influences of the independent variables on responses, and determine the significance of these variables with their optimal values required to achieve the highest yield [8, 11].

Starch, or amylum, is a polymeric carbohydrate composed of several glucose units linked with glycosidic linkages [12]. This polysaccharide is synthesized by most green plants for energy storage [13]. Over the past few decades, two primary themes have dominated scientific research: (i) the creation of novel and influential materials utilizing nanotechnology and (ii) the manufacture of sustainable outputs employing renewable resources [14]. The industrial application of nano-sized starch is prominent in medical, pharmaceuticals, food, and packaging owing to its advantageous properties, including biodegradability, renewability, biocompatibility, non-toxicity, and cost-effectiveness [15, 16]. Starch nanoparticles (StNPs) are characterized as particles with at least one dimension smaller than 1000 nm yet larger than an individual molecule. Furthermore, the literature frequently stipulates additional rigorous size criteria, specifically that at least one dimension must not surpass 300 nm [15]. Over the last decade, StNPs have emerged as a more acceptable and affordable substitute for activated carbon in wastewater treatment [17]. Additionally, they have emerged as an innovative nanocarrier for drug delivery and are increasingly favored as an emulsion stabilizer and fat replacer in the food industry [18]. Instant starches are produced using physical processes utilizing a microwave oven, autoclave, ultrasonicator, drum dryer, extruder, and spray dryer. Surface modification of starch in chemical processes is achieved through oxidation, cationization, acetylation, cross-linking, and alkaline and acidic hydrolysis [19, 20]. Numerous studies have shown that synthesizing StNPs using physical and chemical methods is costly, energy-intensive, and can negatively impact human health and the environment [21, 22]. Moreover, these procedures adversely affect the end products. In contrast to those above physical and chemical methods, the enzymatic synthesis of StNPs exerts milder impacts on the final products, rendering them more environmentally friendly, safe, and healthier [19, 22]. Various investigations have explored the enzymatic generation of StNPs utilizing amylase and pullulanase enzymes [23]. Amylases can produce StNPs with enhanced solubility, gelation, and viscosity characteristics by hydrolyzing soluble starches [24, 25]. A variety of factors, including the kind and quantity of enzyme, the type and amount of starch within solution, the process of gelatinization, and its handling conditions (e.g., centrifugation g-force and duration, microwave power and duration, and autoclave temperature, pressure, and duration), significantly influence the biological and physicochemical characteristics of the resultant StNPs [26].

This study seeks to acquire a novel bacterial isolate that simultaneously produces the α-amylase enzyme and starch nanoparticles (StNPs). The amylolytic isolate was biochemically and subsequently molecularly identified using the sequencing of the 16 S rRNA gene. The yield of α-amylase has been improved through the statistical optimization of culture conditions utilizing the statistical tools such as P-BD and B-BD of response surface methodology (RSM). The StNPs were green-synthesized from bulk starch with three distinct doses of the generated α-amylase. Additionally, physicochemical investigation, encompassing Fourier-transform infrared spectroscopy (FTIR), dynamic light scattering (DLS), and high-resolution transmission electron microscopy (HR-TEM), was utilized for the characterization of StNPs.

## Materials and methods

### Materials

Soluble starch, peptone, DNS (3,5-dinitrosalicylic acid), yeast extract, and agar were provided by Sigma-Aldrich Co. (St. Louis, MO, USA). Magnesium sulfate, beef extract, phenol, and ammonium sulfate were supplied by SDFCL Sd Fine-Chem Limited (Mumbai, India). Potassium sodium tartrate, potassium dihydrogen phosphate, and calcium chloride were obtained from Fluka BioChemica (Buchs, Switzerland). All other chemicals utilized were of analytical-grade quality.

### Isolation of α-amylase-producing bacterial isolate

Soil sample (10 g) collected from agricultural fields in Minya El-Qamh City (30°27’13.2"N 31°17’55.6” E), Sharkiya governorate, Egypt, was suspended into a saline (0.9% NaCl) solution (90 mL), serially diluted (10^− 1^-10^− 6^), and cultivated on nutrient agar plates (pH 7.0) using a spreading manner [27]. After incubation for 24 h at 30 °C, the bacterial colonies were re-grown in nutrient agar slants and maintained for further study at 4 °C.

### Qualitative screening for amylolytic bacterial isolates

The α-amylase activity was qualitatively examined by streaking the bacterial isolates on plates of nutrient agar medium containing 1% soluble starch [9]. These plates were incubated for 48 h at 30 °C and subsequently immersed in a 1% (w/v) iodine solution for staining. The appearance of halo zones surrounding the bacterial colonies identified the amylolytic efficiency of the positive isolates.

### Amylolytic bacterial isolate characterization

Depending on Bergey’s Manual of Determinative Bacteriology [28], the potent isolate MA6 was described according to its morphological and physiological characteristics, which were determined through microscopic examinations and various biochemical tests [27].

### Identification of the amylolytic isolate via the 16 S rRNA gene sequence

The gDNA was extracted after growing the target isolate (MA6) overnight at 30 °C within LB (Luria Bertani) medium based on the protocol of GeneJET gDNA Purification Kit supplied by Thermo-Scientific. The 16 S rRNA gene multiplication was conducted using a polymerase chain reaction (PCR) technique on a thermocycler (Biometra Thermocycler, Germany) with oligonucleotides of 2 global primers: 27 F (5’-GAGTTTGATCCTGGCTCAG-3’) and 1492R (5’-GGTTACCTTGTTACGACTT-3’). The conditions of PCR began with a primary denaturation phase at 94 °C for 4-min. This was followed with 35 runs, each comprising denaturation at 94 °C for 30-sec, annealing at 55 °C for 30-sec, and extension at 72 °C for 60-sec. The process terminated with a final elongation phase at 72 °C for 10-min. Following the protocol outlined in the QIAquick PCR Purification Kit (Qiagen, Germany), the produced bands of PCR were purified and then sent to Macrogen Inc. (Seoul, South Korea) for sequencing via an automated DNA sequencer (ABI 3730 XL). A comparative alignment of the resulting sequence (16 S rRNA gene) with other ones in the National Center for Biotechnology Information (NCBI) was done via the BLAST algorithm (http://www.ncbi.nlm.nih.gov/blast/) online website. Also, the search for sequence homology was carried out in the GenBank (NCBI) nucleotide database to determine the isolate MA6 similarity with other phylogenetic relatives [29]. Moreover, the MEGA 11 software program, based on the neighbor-joining manner [30], was employed to create the phylogenetic tree of the target isolate MA6 [29].

### α-Amylase production by the potent isolate

A loopful of the isolate MA6 was cultivated in a conical flask with 25 mL of sterilized nutrient broth medium/pH 7.0. This flask was left overnight within a shaker of 150 rpm at 30 °C and served as a bacterial inoculum. The α-amylase production was carried out through SmF (submerged fermentation) using a control (basic) medium, which consists of (%): soluble starch, 1; peptone, 0.5; yeast extract, 0.2; KH_2_PO_4_, 0.05; MgSO_4_, 0.05; CaCl_2_, 0.05; NaCl, 0.15 [31]. After sterilizing the previous medium (50 mL, pH 7.0) and cultivating it with the inoculum (1 mL), it was placed within a shaker (150 rpm) for 48 h at 30 °C. Subsequently, the culture was subjected to centrifugation under cooling (4 °C) at 8,000 xg for 20-min and the obtained extract (crude α-amylase) was utilized for enzymatic activity estimation.

### Activity assessment of α-amylase

The combination was formed through mixing 0.5 mL of α-amylase enzyme and 0.5 mL of 1% (w/v) soluble starch/100 mM phosphate buffer (pH 7.0) [32]. This mixture was maintained within a water bath for 30-min under 40 °C, and the method of Miller [33] was used to determine the produced reducing sugar using the DNS (3,5-dinitrosalicylic acid) reagent. To terminate the reaction, 1 mL of the DNS reagent was added to the mixture and boiled for 10-min. Also, a blank (control) sample was prepared by boiling a certain volume (0.5 mL) of crude extract for 20-min to get inactivated (denatured) enzyme, which was added to 0.5 mL of the substrate, and then assessed using DNS method as aforementioned. After cooling, the optical density of the color developed was measured against blank at a wavelength of 540 nm using a spectrophotometer. One unit of enzymatic activity was identified as the enzyme value wanted to liberate 1 µmole of the reduced sugars (glucose units) per minute at the assessment parameters [32].

### α-Amylase production optimization using multi-factorial designs

#### Plackett-Burman design (P-BD)

The P-BD was employed to examine various variables significantly affecting the process [34]. In the current study, 2 levels [low (-), high (+)] of 11 variables (soluble starch, wheat bran, peptone, yeast extract, (NH_4_)_2_SO_4_, MgSO_4_, KH_2_PO_4_, CaCl_2_, incubation time, culture pH, and inoculum size) were evaluated to determine their impact on the productivity of *B. subtilis* strain-MA6 α-amylase. The selection of the variables tested was determined by the biochemical characteristics of the bacterial strain, findings from the literature review, and the fundamental components of the basic (control) medium. Based on P-BD, 12 trials were created using the relation of T = *n* + 1, where T denotes the trial number and n represents the number of variables [35]. All experimental trials were done in triplicate, and the estimated means of α-amylase activities (U/mL) were used as responses. The P-BD model depends on a linear (first-order) equation as follows:

1 $$Y=\beta_{0}+\sum\beta i Xi$$

Where: Y denotes the dependent variable (response), β_0_ represents the intercept of the model, β_i_ indicates the variable estimate (linear coefficient), and X_i_ denotes the independent variable level.

### Box-Behnken design (B-BD)

The B-BD was implemented to optimize the most effective variables screened by the P-BD, establishing the optimal values for every variable to achieve the highest yield of α-amylase enzyme [36]. So, three variables, including soluble starch, MgSO_4_, and incubation time that exhibited a significant effect on α-amylase, were further optimized under three levels: low (-), central (0), and high (+). Depending on B-BD, 14 trials were generated by the combinations of the examined variables, with the corresponding α-amylase activities (U/mL) as the responses. The B-BD relies on a quadratic (second-order) model, which can be expressed via the following equation:

2 $$Y=\beta_{0}+\sum\beta i Xi+\sum\beta ii Xi^{2} +\sum\beta ij Xi Xj$$

Where: Y, the response predicted; β_0_, the intercept term; β_i_, the linear coefficient; β_ii_, the quadratic coefficient; β_ij_, the interaction coefficient; X_i_ and X_j_, the independent variables.

### Characterization of the produced starch nanoparticles (StNPs)

According to the B-BD, trial 5 (B-BD/T_5_), trial 7 (B-BD/T_7_), and trial 13 (B-BD/T_13_) were selected for characterizing the obtained StNPs. The characterization of produced StNPs was carried out using physicochemical measurements, including Fourier-transform infrared spectroscopy (FTIR) spectrometer (Nicolet Impact-400 FT-IR spectrophotometer) in the 400–4000 cm^− 1^ range, Dynamic light scattering (DLS) instrument (Santa Barbara, CA, USA) that was utilized to ascertain the average particle size distribution in nm and average zeta potential in mV, under conditions of 23 °C, with the incident light being the 632.8 nm line of a HeNe laser at an angle of 13.9°. The DLS measurements were conducted in triplicates, and the recorded values were the means with standard deviations (± SD). The topographical analysis included a High-resolution transmission electron microscopy (HR-TEM) Model JEM2010, Japan, which was applied. The obtained images were processed using the free software ImageJ.

### Statistical analysis and software

To assess the significance of the statistical model and each component of the equation, we utilized analysis of variance (ANOVA), which included the *F*-test and probability value (*P*-value). Additionally, the model’s fit was evaluated using the determination coefficient (*R²*) and the Adjusted *R²*. The statistical software Design Expert 13.0 (Stat Ease Inc., Minneapolis, MN, USA) was employed for the design of experiments, as well as for the analysis and interpretation of the experimental results [37]. All experiments were performed in 3 replicates, and the results presented are the means ± standard deviations (± SD).

## Results and discussion

### Isolation and qualitative investigation of amylolytic bacterial isolates

**Fig. 1:**
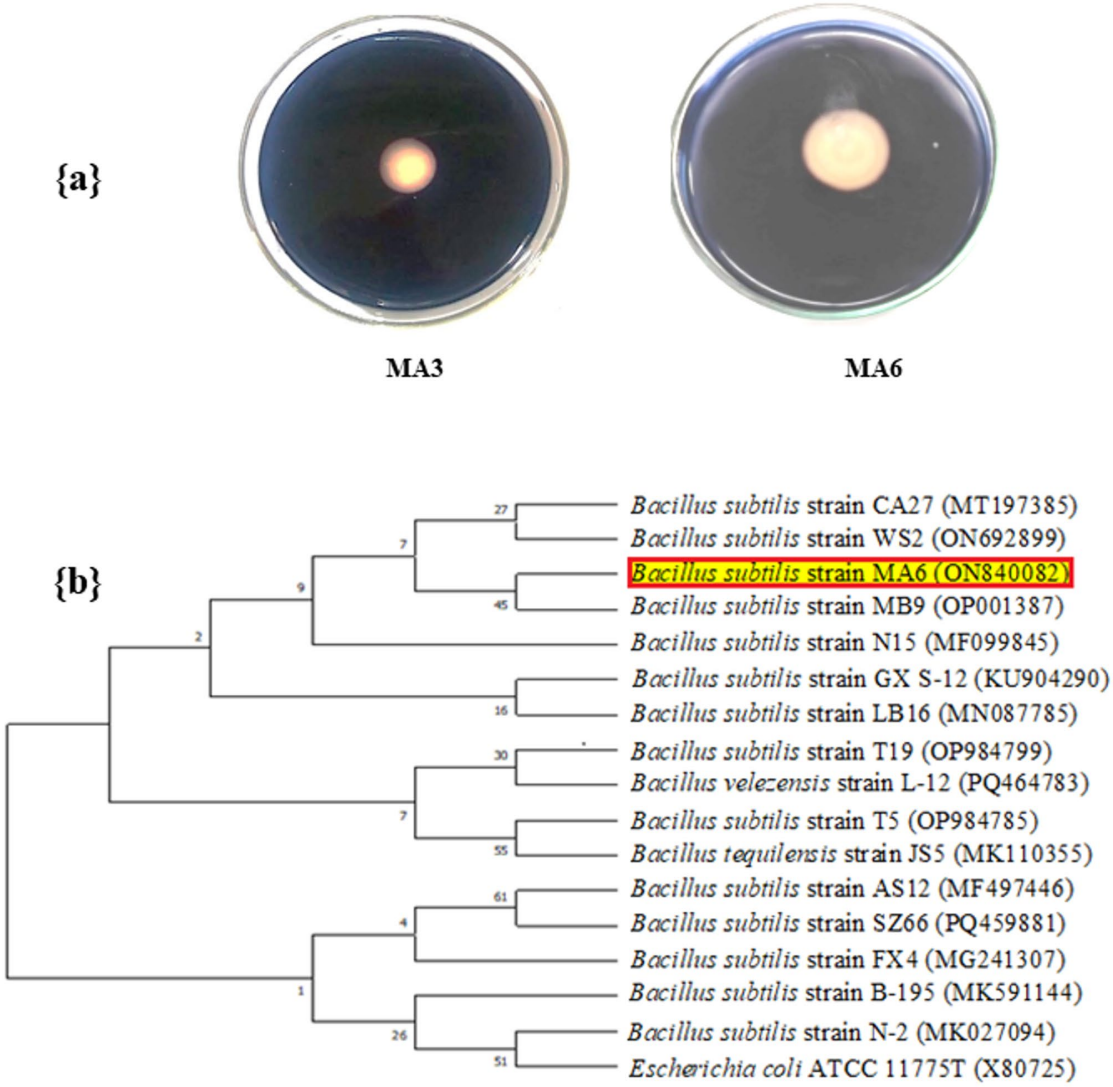
(a) Amylolytic activity of the isolates MA3 and MA6 showing clear zones on starch-agar plates (b) *B. subtilis* strain-MA6 (ON840082) phylogenetic tree, depending on the sequence of 16 S rRNA gene alignments with other relatives inside the GenBank/NCBI. The out-group reference strain was *E. coli* ATCC 11775T (X80725)

The isolation step revealed twenty-four (24) pure bacterial colonies that were qualitatively examined to produce α-amylase by growing them upon starch-agar plates immersed in 1% (w/v) iodine solution. After investigation, two (2) isolates coded as MA3 and MA6 showed an amylolytic activity that was distinguished by a clear zone formation surrounding each colony at a dark-blue background due to starch molecule degradation by the action of α-amylase enzyme as seen in Fig. 1a. Based on the zone of clearance diameter, the bacterial isolate MA6 which has a more expansive clear zone around its colony was selected for further considerations.

### Characterization of the potent amylolytic isolate

After characterizing the potent isolate MA6 biochemically and morphologically depending on Bergey’s Manual of Determinative Bacteriology methods [28], it was described as a rod-shaped, spores-forming, aerobic, motile, and gram-positive bacterium. Moreover, the results of biochemical tests were illustrated as follows: catalase (+), oxidase activity (+), methyl red (-), urease activity (-), indole (-), nitrate reduction (+), citrate utilization (+), glucose fermentation (+), maltose fermentation (+), sucrose fermentation (+), starch hydrolysis (+), gelatin hydrolysis (+), casein hydrolysis (+), CMC hydrolysis (+), and LBG (locust bean gum) hydrolysis (-).

### Identification of the potent amylolytic isolate molecularly

The sequence of 16 S rRNA gene, indicating the chosen (potent) isolate, was determined and submitted into the GenBank/NCBI database library under the designation *Bacillus subtilis* strain-MA6 (accession number: ON840082). The resemblance searching for *B. subtilis* strain-MA6 exhibited 99.4% homogeneity with the nearest relatives in the NCBI. Further, the *B. subtilis* strain-MA6 (ON840082) phylogenetic tree generated using MEGA 11 software based on the 16 S rRNA gene sequence alignments with other relatives inside the GenBank/NCBI was displayed in Fig. 1b.

### α-Amylase production optimization using multi-factorial designs

#### Plackett-Burman design (P-BD)

The P-BD is the first statistical step to identify the most critical parameters for enhancing α-amylase productivity from *B. subtilis* strain-MA6 [34]. It allows the screening of 11 independent variables (Table 1) at 2 levels [low (-), high (+)], and the diverse combination between these variables produced 12 trials that exhibited significant variance in the response (α-amylase activity) as shown in Table 2. The highest yield of α-amylase was observed at trial 10 (630.6 U/mL), which was greater than that acquired from the original (basic) medium (59.5 U/mL) by 10.6-fold.

**Table 1:** Different levels of various variables tested by the P-BD

Variable		Unit	Level	
Name	Code		Low [−]	High [+]
Soluble starch	A	% (w/v)	0.3	1
Wheat bran	B	% (w/v)	0	0.5
Peptone	C	% (w/v)	0.2	0.5
Yeast extract	D	% (w/v)	0.1	0.3
(NH_4_)_2_SO_4_	E	% (w/v)	0	0.2
MgSO_4_	F	% (w/v)	0.05	0.2
KH_2_PO_4_	G	% (w/v)	0.05	0.2
CaCl_2_	H	% (w/v)	0.025	0.1
Culture pH	J	-	6	8
Incubation time	K	h	48	72
Inoculum size	L	% (v/v)	1	2

**Table 2:** Screening of various variables influencing α-amylase production using P-BD

Trial	A	B	C	D	E	F	G	H	J	K	L	α-Amylase activity	Predicted values
	%	%	%	%	%	%	%	%	-	h	%	U/mL	U/mL
1	1	0	0.2	0.1	0.2	0.05	0.2	0.1	6	72	2	223.3	216.12
2	1	0.5	0.5	0.1	0	0.05	0.2	0.025	8	72	1	292	299.18
3	1	0	0.5	0.3	0	0.2	0.2	0.1	6	48	1	348.5	341.32
4	1	0	0.5	0.3	0.2	0.05	0.05	0.025	8	48	2	373	377.10
5	0.3	0	0.2	0.1	0	0.05	0.05	0.025	6	48	1	581.6	585.70
6	0.3	0	0.5	0.1	0.2	0.2	0.05	0.1	8	72	1	616	608.82
7	0.3	0.5	0.2	0.3	0.2	0.05	0.2	0.1	8	48	1	56.5	52.40
8	0.3	0.5	0.5	0.1	0.2	0.2	0.2	0.025	6	48	2	222.1	229.28
9	0.3	0	0.2	0.3	0	0.2	0.2	0.025	8	72	2	171.8	175.90
10	1	0.5	0.2	0.3	0.2	0.2	0.05	0.025	6	72	1	630.6	637.78
11	1	0.5	0.2	0.1	0	0.2	0.05	0.1	8	48	2	598.8	594.70
12	0.3	0.5	0.5	0.3	0	0.05	0.05	0.1	6	72	2	495.7	491.60

Multiple-regression analyses were done to show the effectiveness of the examined variables on the α-amylase yield from *B. subtilis* strain-MA6, and the P-BD data were demonstrated through the resulting first-order (linear) model of the following equation:

3 $$ \begin{aligned} {\text{Y}}\, = & \,{\text{384}}.{\text{16}}\, + \,{\text{26}}.{\text{88A}}\, + \,{\text{7}}.0{\text{6C }} - {\text{38}}.{\text{14D }} - {\text{3}}0.{\text{57E}}\, \\ & + \,{\text{47}}.{\text{14F }} - {\text{165}}.{\text{13G }} - {\text{32}}.{\text{81J}}\, + \,{\text{2}}0.{\text{74K }} - {\text{36}}.{\text{71L}} \\ \end{aligned} $$

Where: Y, α-amylase activity U/mL (response predicted); A, soluble starch; C, peptone; D, yeast extract; E, (NH_4_)_2_SO_4_; F, MgSO_4_; G, KH_2_PO_4_; J, culture pH; K, incubation time; L, inoculum size.

The ANOVA (analysis of variance) showed the model’s efficiency and illustrated which independent variables significantly affected α-amylase productivity. As seen in Table 3, both *F*-value (230.66) and *P*-value (0.0043) indicate that the P-BD was a significant model. Also, the lower *P*-values (*P*-values < 0.05) point to the significance of the corresponding model terms. From Table 3, the critical variables were soluble starch, yeast extract, (NH_4_)_2_SO_4_, MgSO_4_, KH_2_PO_4_, incubation time, culture pH, and inoculum size. Moreover, the fitness and eligibility of the P-BD could be verified via the *R*^*2*^ (determination coefficient) value, where the model with an *R*^*2*^-value close to 1 (*R*^*2*^-value > 0.9) demonstrates a strong correlation between the recorded and predicted results [29]. According to Table 3, the *R*^*2*^-value of 0.9990 means the statistical design was efficient and can interpret 99.90% of the response variation (α-amylase activities). Also, the Adjusted *R*^*2*^-value (0.9947) and the Predicted *R*^*2*^-value (0.9654) highlight the P-BD strength and prove the great alignment among the predicted and actual values. Additionally, the lower coefficient of variation value (CV-value = 3.73%) implies that the model has high reliability and the experimental performance has excellent precision.

**Table 3:** ANOVA of P-BD showing the model and tested variables significance

Source	Sum of Squares	DF	Mean Square	SD	F-value	*P*-value	
Model	4.261E + 05	9	47339.21	14.33	230.66	0.0043	Significant
A-Soluble starch	8667.19	1	8667.19	0.3656	42.23	0.0229	
C-Peptone	597.84	1	597.84	0.1567	2.91	0.2300	
D-Yeast extract	17457.44	1	17457.44	0.1044	85.06	0.0116	
E-(NH_4_)_2_SO_4_	11217.97	1	11217.97	0.1044	54.66	0.0178	
F-MgSO_4_	26668.04	1	26668.04	0.0783	129.94	0.0076	
G-KH_2_PO_4_	3.272E + 05	1	3.272E + 05	0.0783	1594.28	0.0006	
J-Culture pH	12916.64	1	12916.64	1.04	62.94	0.0155	
K-Incubation time	5162.60	1	5162.60	12.53	25.16	0.0375	
L-Inoculum size	16170.02	1	16170.02	0.5222	78.79	0.0125	
Residual	410.46	2	205.23				
Cor Total	4.265E + 05	11					

*R²* **=** 0.9990, Adjusted *R*^*2*^ = 0.9947, Predicted *R*^*2*^ = 0.9654, CV = 3.73%, Adequate Precision = 44.7620

DF (degree of freedom), SD (standard deviation), Significant (*P* < 0.05), Un-significant (*P* > 0.05)

**Fig. 2:**
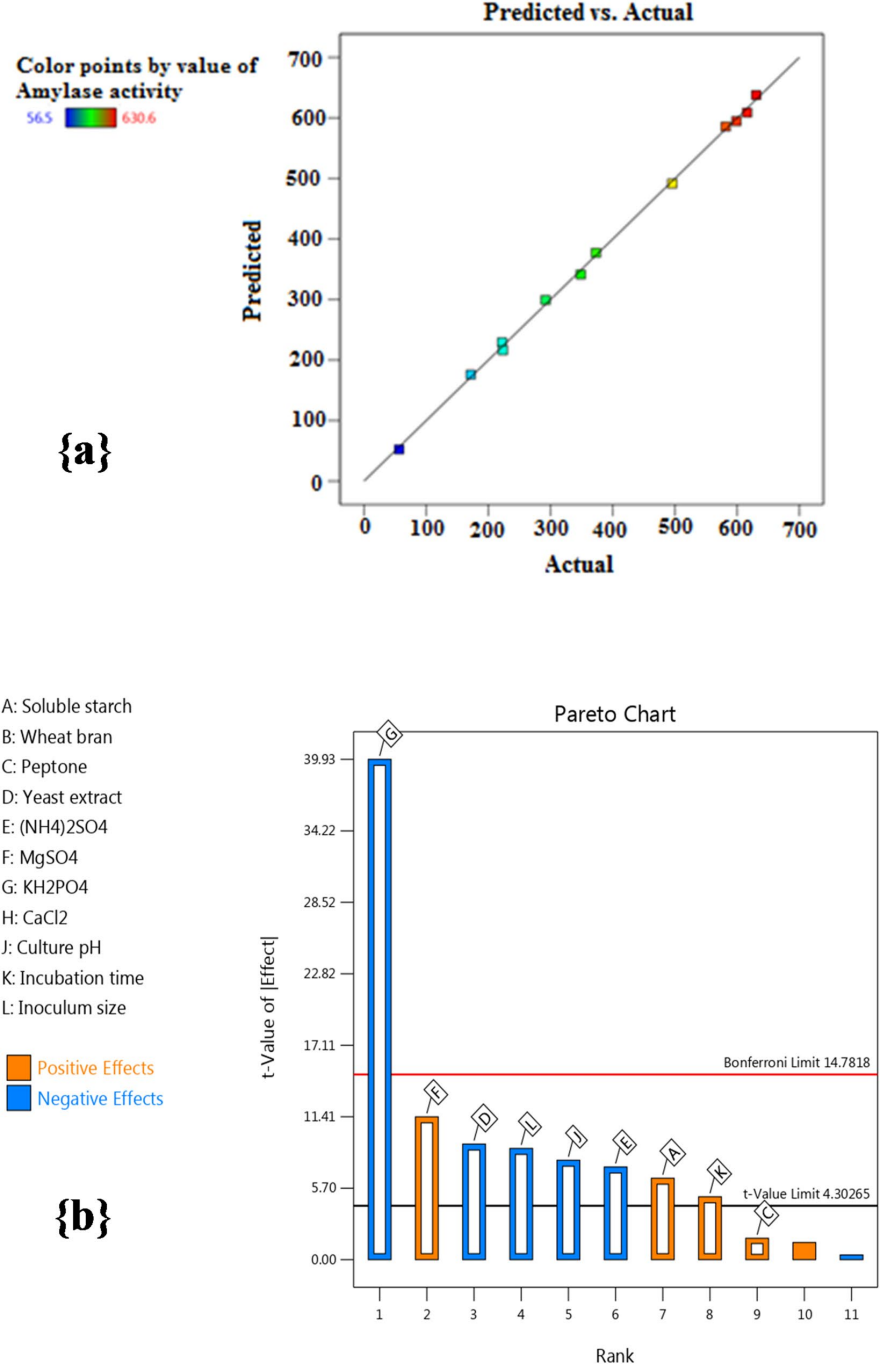
(a) The relevance among predicted vs. actual α-amylase activity values of P-BD (b) The descending order of significant variables displayed by the Pareto plot of P-BD. The “Orange color” indicated for “Positive effect”, while the “Blue color” indicated for “Negative effect” of the tested variables on α-amylase activity

The relevance among the actual and predicted activities (responses) values (Fig. 2a) displayed the extreme closure amidst these values, indicating the model’s importance and ability to illustrate the statistical data. Conversely, the descending order of the screened variables displayed by the Pareto Plot of P-BD (Fig. 2b) shows the comparative significance of each tested variable and its effect on the response. As seen in Fig. 2b, the variables that exhibited the highest level of significance were MgSO_4_, soluble starch, and incubation time, which affected α-amylase activity positively, whereas KH_2_PO_4_, yeast extract, inoculum size, culture pH, and (NH_4_)_2_SO_4_ affected α-amylase activity negatively. Likewise, Hallol et al. [7] suggested that the significant factors, including peptone and incubation period, positively impacted the α-amylase yield. At the same time, aeration and CaCl_2_ revealed a negative effect. Also, P-BD can be used to improve amylase production by *Bacillus* sp. H7, which confirmed that starch, incubation period, and medium pH were significant variables, but yeast extract, KH_2_PO_4_, (NH_4_)_2_SO_4_, and inoculum concentration were non-significant ones [9]. Furthermore, incubation time, CaCl_2_, and pH were identified as having a significant positive influence on α-amylase productivity, as Abdullah et al. [5] reported.

### Box-Behnken design (B-BD)

Based on P-BD results, the most effective variables (soluble starch, MgSO_4_, and incubation time) were further studied using B-BD to obtain adequate levels (concentrations) for enhancing the *B. subtilis* strain-MA6 α-amylase production. The experimental trials (14 trials) generated by the B-BD model and their corresponding responses (α-amylase activities U/mL) were presented in Table 4.

**Table 4:** B-BD trials including variables selected for α-amylase production optimization

Trial	A: Soluble starch	B: MgSO_4_	C: Incubation time	α-Amylase activity	Predicted values
	% (w/v)	% (w/v)	h	U/mL	U/mL
1	(+) 2	(-) 0.2	(0) 96	460	478.20
2	(0) 1.25	(-) 0.2	(+) 120	609.2	588.48
3	(+) 2	(0) 0.4	(-) 72	511.6	491.58
4	(0) 1.25	(+) 0.6	(-) 72	404.9	425.63
5	(0) 1.25	(-) 0.2	(-) 72	552.1	553.93
6	(0) 1.25	(0) 0.4	(0) 96	430.6	426.15
7	(+) 2	(+) 0.6	(0) 96	303.7	303.00
8	(-) 0.5	(-) 0.2	(0) 96	671.8	672.50
9	(+) 2	(0) 0.4	(+) 120	368.1	370.63
10	(-) 0.5	(+) 0.6	(0) 96	717.8	699.60
11	(-) 0.5	(0) 0.4	(-) 72	579.8	577.28
12	(0) 1.25	(+) 0.6	(+) 120	570.5	568.68
13	(-) 0.5	(0) 0.4	(+) 120	855.8	875.83
14	(0) 1.25	(0) 0.4	(0) 96	421.7	426.15

After a comprehensive statistical analysis of the experimental data, the B-BD results can be explained by the quadratic equation as follows:

4 $$ \begin{aligned} {\text{Y }}\left( {{\text{U}}/{\text{mL}}} \right) = & {\text{426}}.{\text{15 }} - {\text{147}}.{\text{7A}} - {\text{37}}.0{\text{3B}} + {\text{44}}.{\text{4C }} \\ & - {\text{5}}0.{\text{57AB}} - {\text{1}}0{\text{4}}.{\text{88AC }} + {\text{27}}.{\text{1BC }} \\ & + {\text{78}}.{\text{41A}}{\text{ }} + {\text{33}}.{\text{76B}}{\text{ }} + {\text{74}}.{\text{26C}} \\ \end{aligned} $$

Where: Y, α-amylase activity U/mL (predicted response); A, soluble starch; B, MgSO_4_; and C, incubation time.

The ANOVA of B-BD was used to identify the model’s strength and the significance of the equation terms. From Table 5, the model’s *P*-value (lower than 0.05) and *F*-value (54.04) refer to the significance of the B-BD [38]. The data exhibited a high variation in the predicted response, which the model equation could explain. Moreover, the values of *R*^*2*^ (0.9918), Adjusted *R*^*2*^ (0.9735), and Predicted *R*^*2*^ (0.8711) implied the fitness of the model and demonstrated the strong accordance between the recorded and expected values. Depending on the *R*^*2*^-value, the statistical design can explain 99.18% of the overall divergences in the enzyme activity, and only 0.82% of these divergences are not explained [38].

Furthermore, adequate precision, which is affected by the signal-to-noise ratio, is considered sufficient when this ratio exceeds 4. Our design’s adequate precision value was 27.7655, indicating a proper signal and confirming that the model was suitable for effectively exploring and optimizing the design space [39]. Also, the CV-value of 4.58% (lower value) provided a good precision and accuracy for the executed experiments. In addition, the insignificant lack of fit (*P*-value = 0.1637) indicated that the regression model equation was adequate for predicting the responses [40].

**Table 5:** ANOVA of the B-BD to optimize α-amylase production

Source	Sum of Squares	DF	Mean Square	SD	F-value	*P*-value	
Model	2.898E + 05	9	32204.41	24.41	54.04	0.0008	Significant
A- Soluble starch	1.746E + 05	1	1.746E + 05	0.5883	292.98	< 0.0001	
B MgSO_4_	10966.81	1	10966.81	0.1569	18.40	0.0127	
C- Incubation time	15770.88	1	15770.88	18.83	26.47	0.0068	
AB	10231.32	1	10231.32		17.17	0.0143	
AC	43995.06	1	43995.06		73.83	0.0010	
BC	2943.06	1	2943.06		4.94	0.0904	
A²	19675.26	1	19675.26		33.02	0.0045	
B²	3647.70	1	3647.70		6.12	0.0686	
C²	17647.74	1	17647.74		29.62	0.0055	
Residual	2383.53	4	595.88				
Lack of Fit	2343.93	3	781.31		19.73	0.1637	Non-significant
Pure Error	39.61	1	39.61				
Cor Total	2.922E + 05	13					

*R²* **=** 0.9918, Adjusted *R*^*2*^ = 0.9735, Predicted *R*^*2*^ = 0.8711, CV = 4.58%, Adequate Precision = 27.7655

DF (degree of freedom), SD (standard deviation), Significant (*P* < 0.05), Non-significant (*P* > 0.05)

Moreover, the coefficient estimates, *P-*values, standard errors, and confidence intervals (CI) of model terms (linear, quadratic, and interaction) were illustrated in Table 6. The coefficient estimate represents the expected change in response per unit change in factor value when all remaining factors are held constant. The intercept reflects the overall average response across all experimental runs in an orthogonal design. The coefficients adjust this average based on the specific factor settings. When factors are orthogonal, the Variance Inflation Factors (VIFs) equal 1; VIFs exceeding 1 suggest the presence of multicollinearity, with higher VIF values indicating a stronger correlation among factors. Generally, VIFs below 10 are considered acceptable.

**Table 6:** Coefficient estimates, standard errors, *P-*values and confidence intervals (CI) of B-BD

Term	Coefficient estimate	Standard error	*P*-value	95% CI Low	95% CI High	VIF
Intercept	426.15	17.26		378.23	474.07	
A- Soluble starch	-147.73	8.60	< 0.0001	-171.61	-123.84	1.0000
B MgSO_4_	-37.03	8.60	0.0127	-60.91	-13.14	1.0000
C- Incubation time	44.40	8.60	0.0068	20.52	68.28	1.0000
AB	-50.57	12.16	0.0143	-84.35	-16.80	1.0000
AC	-104.88	12.16	0.0010	-138.65	-71.10	1.0000
BC	27.12	12.16	0.0904	-6.65	60.90	1.0000
A²	78.41	13.60	0.0045	40.15	115.67	1.07
B²	33.76	13.60	0.0686	-4.50	71.02	1.07
C²	74.26	13.60	0.0055	36.00	111.52	1.07

**Fig. 3:**
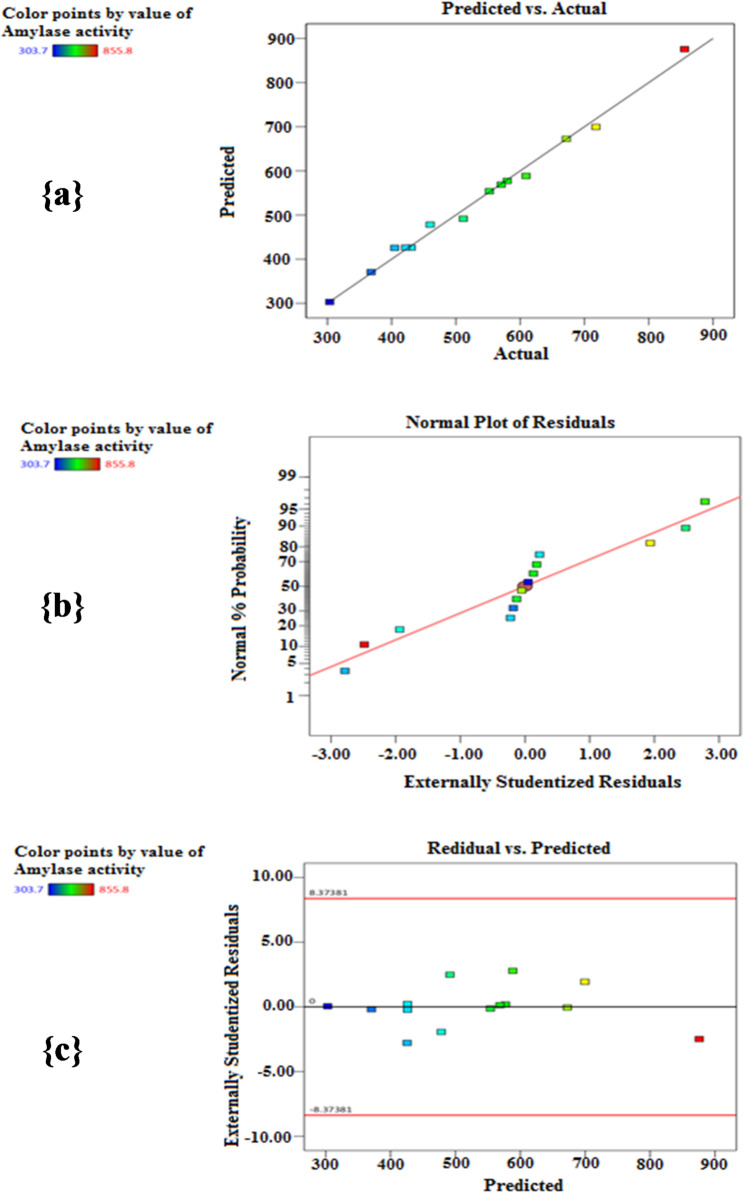
B-BD of α-amylase production showing (a) the relevance among actual and predicted results, (b) the plot of normal probability for studentized residuals, (c) the graph of residuals against predicted results

On the other hand, the model’s fit quality was confirmed through the high closure among the predicted and actual responses (Fig. 3a). Moreover, the plot of normal probability for studentized residuals revealed that the plotted points were near a straight line (Fig. 3b) indicating that this design was suitable for illustrating the production of *B. subtilis* strain-MA6 α-amylase. Further, the random spreading of residuals close to a horizontal zero line was shown by the graph of residuals against predicted results (Fig. 3c), which pointed to the fitness of the statistical model [38].

**Fig. 4:**
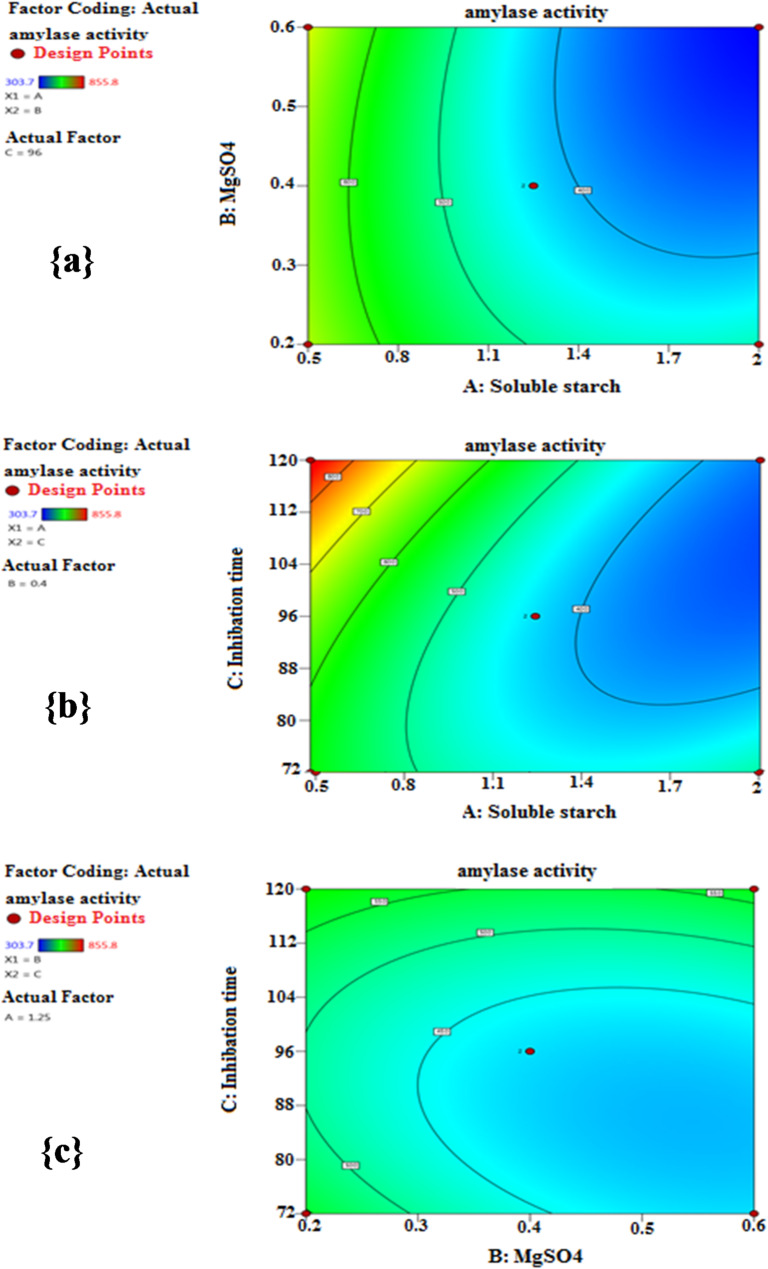
Contour plots of response surface illustrating the influence of the interacted variables on α-amylase activity (a) soluble starch and MgSO_4_, (b) soluble starch and incubation time, (c) MgSO_4_ and incubation time

The response surface contour plots (Fig. 4) reflect a visual elucidation of the interacted variables to identify the best levels affecting response, where the activity of α-amylase was plotted on the z-axis versus each of the two variables, maintaining the other one at its (0) level. As seen in Fig. 4a, the interaction between soluble starch and MgSO_4_, while incubation time was kept at its central level (96 h), demonstrated a maximal α-amylase yield (717.8 U/mL) at 0.5% (low level) of soluble starch and 0.6% (high level) of MgSO_4_. Additionally, Fig. 4b displays the interaction effect of soluble starch and incubation time on α-amylase activity, conserving MgSO_4_ at its central level (0.4%). In this case, the greatest α-amylase productivity (855.8 U/mL) was achieved at 0.5% (low level) of soluble starch and 120 h (high level) of incubation time. Furthermore, Fig. 4c shows the interaction between MgSO_4_ and incubation time, whereas soluble starch was fixed at the central point (1.25%). The results indicated that the maximum α-amylase activity (609.2 U/mL) occurred at 0.2% (low level) of MgSO_4_ and 120 h (high level) of incubation time.

### Model confirmation and validation

**Fig. 5:**
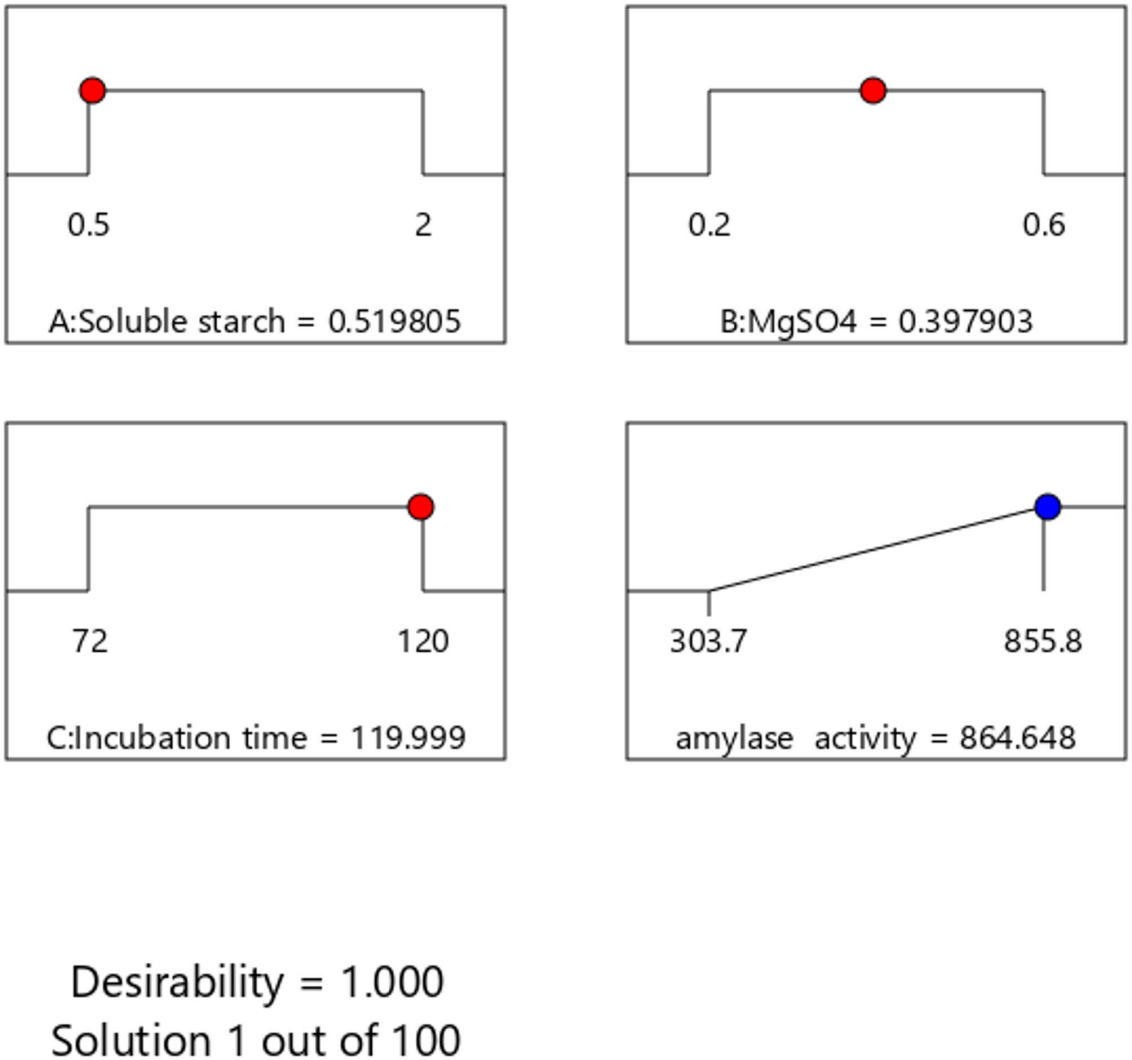
Desirability ramps for α-amylase production optimization

A confirmatory experiment has been implemented according to the optimal values of the examined factors proposed from the model to verify the eligibility and validity of the statistical analysis. As shown in the desirability ramps (Fig. 5), the best levels suggested by the model were soluble starch (0.52%), MgSO_4_ (0.4%), and incubation time (120 h) that predicted a maximum value (864.65 U/mL) of α-amylase activity. The value of α-amylase activity obtained by the laboratory trial was 861.2 U/mL, which agreed with the expected level (864.65 U/mL) by 99.6%, confirming the model validation, the outstanding reliability of the results, and the presence of optimum points.

In the present work, the optimized medium (soluble starch 0.52%, peptone 0.5%, yeast extract 0.1%, (NH_4_)_2_SO_4_ 0.1%, MgSO_4_ 0.4%, KH_2_PO_4_ 0.025%, CaCl_2_ 0.025%, NaCl 0.15%, at pH 6, and incubation time 120 h) using B-BD revealed a 14.5-fold increase in α-amylase production (861.2 U/mL) by *B. subtilis* strain-MA6 compared to the production (59.5 U/mL) from the non-optimized (initial) one. The previous studies investigated by Mostafa et al. [41] and Ousaadi et al. [42] recorded that the α-amylase production by *Bacillus spp. NRC1* and *Streptomyces sp.*, using a statistical design approach, was promoted by 3.11-fold and 4-fold, respectively. Also, Ugwuoji et al. [43] suggested that the use of statistical optimization (RSM) for medium conditions, led to a 2.1-fold increase in the α-amylase production by *Paenibacillus lactis* strain OPSA3. Furthermore, the production of α-amylase from *Bacillus* strain increased by 6.5-fold after optimizing the medium composition using statistical methods (RSM), according to Mabrouk et al. [44].

### Characterization of the produced starch nanoparticles (StNPs)

Based on the B-BD results, trial 5 (B-BD/T5) = α-amylase activity of 552.1 U/mL, trial 7 (B-BD/T7) = α-amylase activity of 303.7 U/mL, and trial 13 (B-BD/T13) = α-amylase activity of 855.8 U/mL, which contained medium (1.25%), high (2%), and low (0.5%) native starch levels, respectively, were selected for the StNPs characterization.

### FTIR

**Fig. 6:**
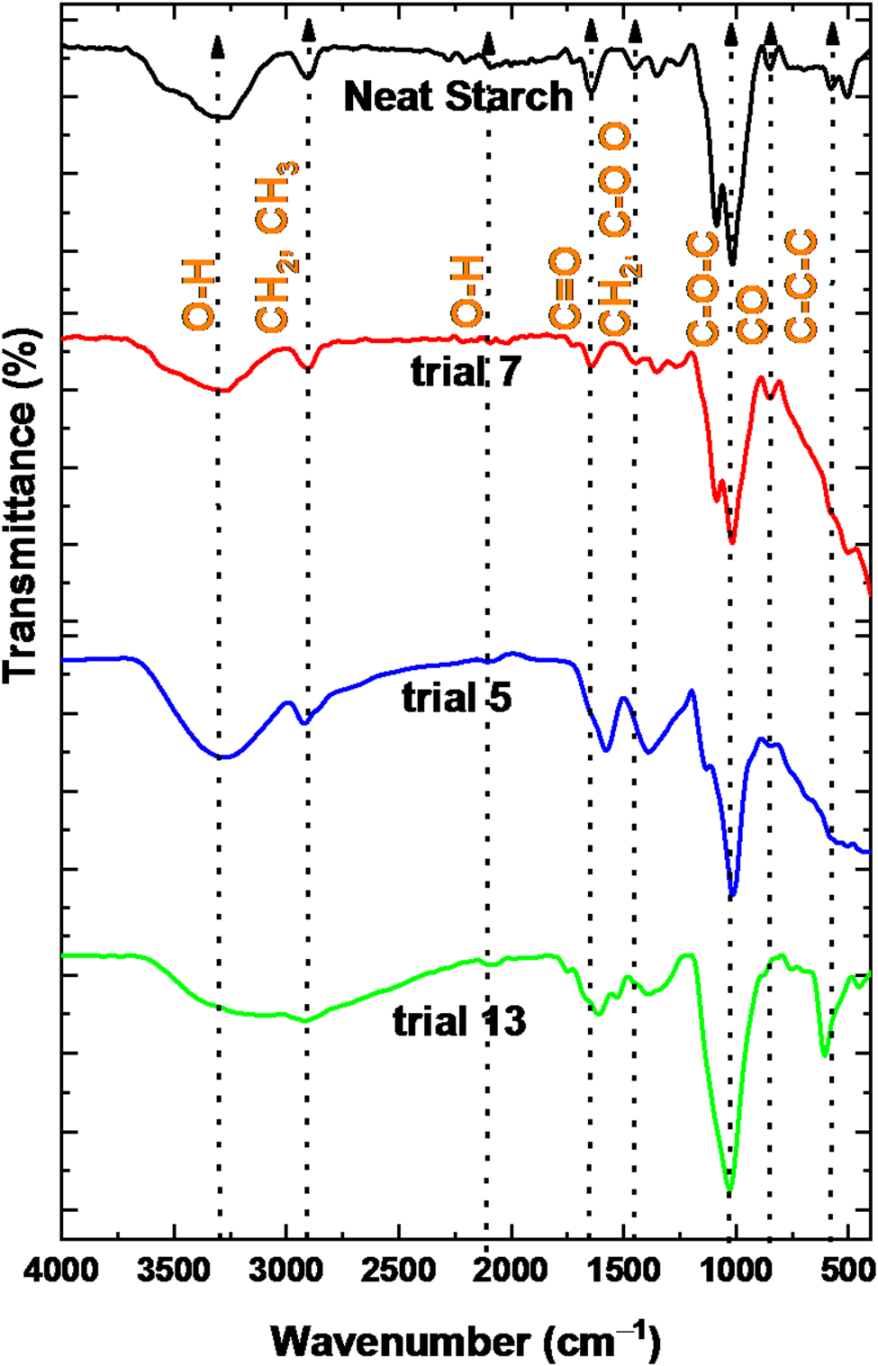
FTIR of native starch and different trials of α-amylase treated starch samples

The FTIR spectrum analysis indicates that specific wavenumbers correspond to unique chemical vibrations related to the composition of starch samples, as presented in Fig. 6. Native starch presented characteristic bands at 3281, and 2893 cm^˗1^ corresponding to O–H stretching vibrations of hydroxyl groups in polysaccharides and vibrations associated with C–H stretching in aliphatic hydrocarbons may result from the presence of non-bound water molecules. The stretching vibration of C = O arises from the carbonyl group assigned at 2091 cm^˗1^ [45]. Additionally, the band at 1634 cm^˗1^ is associated with the deformation vibrations of locally symmetric groups, such as CH_2_ and C-OO in carbohydrates [46]. Moreover, the bands at 1014, 851, and 574 cm^˗1^ were related to the C–O–C stretching vibration of the glycosidic linkage in carbohydrates, which is characteristic of starches and indicative of glycosidic bonds connecting individual glucose units within the starch molecule, the C–O stretching vibrations of the glycosidic linkage in amylose and amylopectin, and the bending vibrations of the C–C–C linkage within the glycosidic bond of carbohydrates, respectively [47, 48]. On the other hand, the treated starch samples with different amounts of α-amylase enzyme via suspected trials 5, 7, and 13 show different effects on the molecular structure. The sample treated with low α-amylase activity (trial 7) showed no significant changes compared to the native starch spectrum. However, the sample treated with medium α-amylase activity (trial 5) shows liberation of the CH_3_ and CH_2_ bands according to the reorganization of the starch chains in the nanoform [49]. The sample treated with high α-amylase activity (trial 13) shows particular changes in the characteristic bands of the native starch. Band of OH, CH_3_, COC, and C–C–C positions were shifted to 3300, 2909, 1022, and 608 cm^˗1^ [50]. Moreover, the band position of C = O was turned to low frequency and assigned at 1617 cm^˗1^ [51]. These outcomes indicate that the free hydrogens become less tightly bound [52, 53]. The inter-strand and inter-double helix hydrogen bonds exhibited retrogradation; helices coalesced to establish a crystalline structure [49, 54].

### DLS

**Fig. 7:**
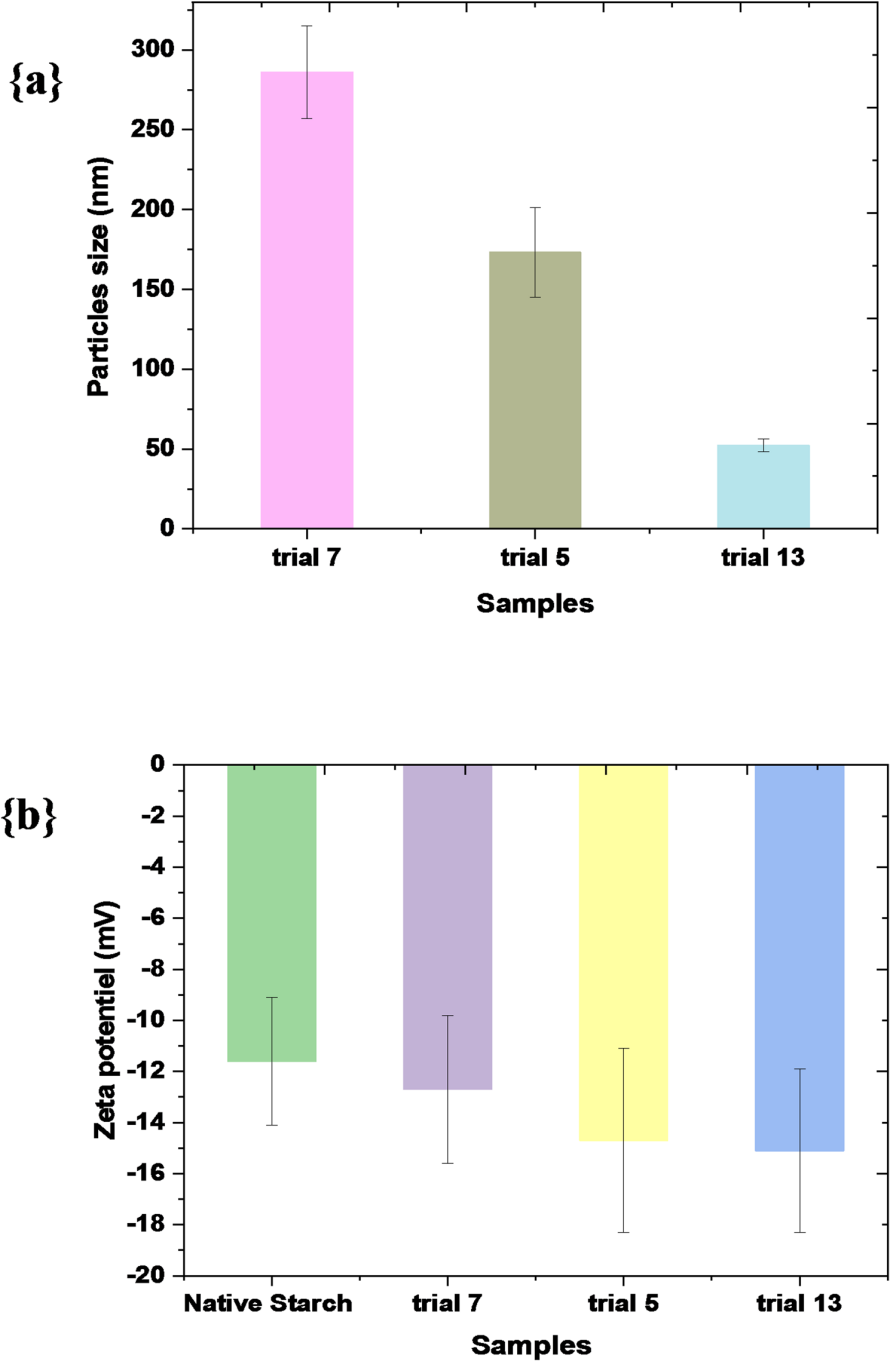
Particle size distribution (a) and average zeta potential (b) of starch samples

Figure 7 presents the DLS, including particle size distribution (nm) and average zeta potential (mV). The particle size of native starch was recorded as 3204 nm (data not shown), reduced to 286.2 ± 29, 173.2 ± 28, and 52.4 ± 4 nm after treatment with low, medium, and high α-amylase activity, respectively (Fig. 7a). On the other side, the average zeta potential was recorded in Fig. 7b. The native starch recorded average zeta potential as -11.6 ± 2.5 mV. However, the different trials of α-amylase-treated samples recorded − 12.7 ± 2.9, -14.7 ± 3.1, and − 15.1 ± 3.2 mV, respectively. The correlation between particle size and average zeta potential measurements was noticed. The decrease in the sample particle size reflects the increase in zeta potential value [55]. The reduction in particle size correlated with the average zeta potential according to the colloidal stability, which reflected a high zeta potential value [56]. This outcome indicates that the free hydrogens become less tightly bound to the energy and distance of the hydrogen bond in starch. The distance between the inter-strand and inter-double helix hydrogen bonds negatively correlated with storage duration. During retrogradation, helices coalesced to form a crystalline structure [57, 58]. These observations confirmed that the α-amylase activity interacted with the starch helix to reduce the particle size. The physicochemical analysis, including FTIR and DLS measurements, emphasized the molecular structure change and stability of the sample treated with high α-amylase activity.

### HR-TEM

**Fig. 8:**
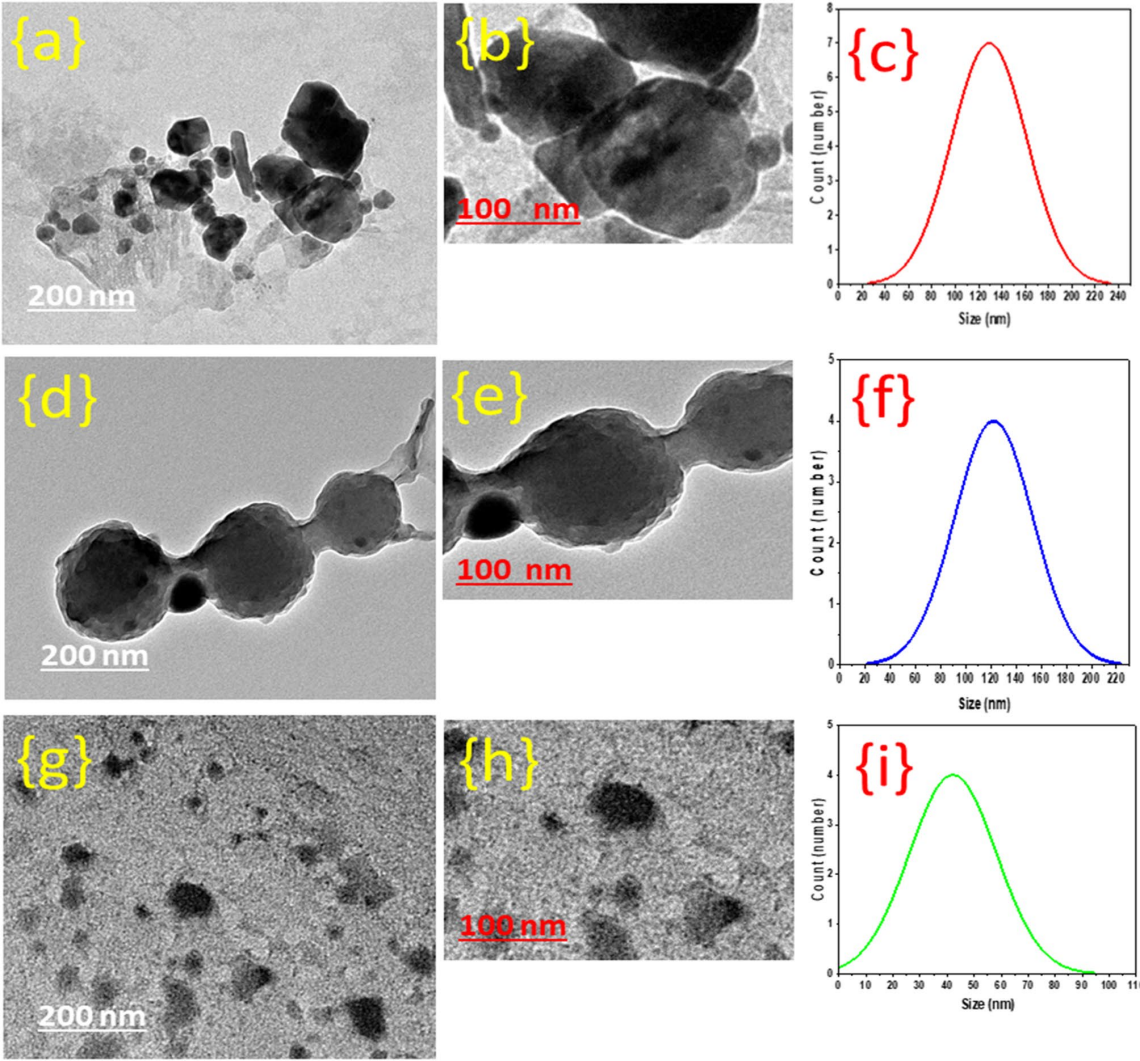
TEM images of the treated starch sample with different concentrations of α-amylase with low and high magnifications [trial 7 (a and b), trial 5 (d and e), and trial 13 (g and h)]. The ImageJ process particle sizes were presented as c, f, and i, respectively

The particle morphological analysis via HR-TEM is shown in Fig. 8. The HR-TEM images of the low α-amylase activity sample (trial 7) in low and high magnifications are presented in Fig. 8a and b, respectively. The sample treated with trail 7 condition presented starch particles as clusters in low magnification image (Fig. 8a); this clustered structure was illustrated as collapsed particles aggregated to each other in high magnification image (Fig. 8b) in an irregular spherical shape with an average particle size of around 135 nm (Fig. 8c). The sample treated with trail 5 condition (the medium α-amylase activity) presented starch particles in a low magnification image as low condensed particles (Fig. 8d). These particles were clearly observed as connected particles to each other in a high magnification image (Fig. 8e) in a spherical shape with an average particle size around 123 nm (Fig. 8f). The high α-amylase treatment samples (trial 13) were presented in low magnification (Fig. 8g) and high magnification image (Fig. 8h) as nanoparticles with spherical morphology, with average particle diameters ranging around 43 nm (Fig. 8i). The trials illustrated a typical StNPs morphology with different particle size according to previous observations [59–61]. Moreover, the StNPs morphology and size emphasized the significant efficiency of α-amylase activity, which is reduced in size with high enzyme activity [22, 23].

## Conclusion

A newly α-amylase producing bacterium was isolated, characterized, and identified as *B. subtilis* strain-MA6 (accession number: ON840082) in the GenBank/NCBI database. In this context, StNPs were produced in situ from bulk starch used as a substrate for enzyme production. In situ production offered an economic and sustainable method according to tools and processing of production as well. Medium conditions were statistically optimized using a multi-factorial design (P-BD and B-BD) approach. MgSO_4_, soluble starch, and incubation time were considered the three essential factors examined by P-BD that positively impacted α-amylase production. The ANOVA outcomes of both P-BD and B-BD, including *F*-value, *P*-value, *R*^*2*^-value, Adjusted *R*^*2*^-value, Predicted *R*^*2*^-value, and CV-value, confirmed the significance (fitness) of the statistical models and reliability of the experiments. The statistically optimized medium using P-BD and B-BD of RSM enhanced α-amylase productivity by 14.5-fold compared to the unoptimized one. Three trials were selected to study the effect of different levels of enzyme concentration. The physicochemical analysis and HR-TEM were applied to the StNPs characterization. In addition, the concentration of the α-amylase enzyme plays a role in converting bulk starch to nanosized particles, affecting the stability of the produced nanoparticles and their size. A high amylase concentration (trial 13) also produced high stability with a small size. It was observed that the factors influencing the particle size reduction are directly linked to the concentration ratio of native starch in relation to the productivity of α-amylase. This observation offered an optimistic technique to produce StNPs via a green and ecofriendly process.

## Data Availability

No datasets were generated or analysed during the current study.
